# Characterization and Performance Analysis of Hydrolyzed versus Non-Hydrolyzed Poly(NVF-co-HEA) Hydrogels for Cosmetic Applications

**DOI:** 10.3390/gels10050311

**Published:** 2024-05-02

**Authors:** Maytinee Yooyod, Thanyaporn Pinthong, Sararat Mahasaranon, Jarupa Viyoch, Sukunya Ross, Gareth M. Ross

**Affiliations:** 1 Biopolymer Group, Department of Chemistry, Faculty of Science, Naresuan University, Phitsanulok 65000, Thailand; maytineey60@nu.ac.th (M.Y.); thanyapornpi62@nu.ac.th (T.P.); 2 Biopolymer Group, Department of Chemistry, Center of Excellence in Biomaterials, Faculty of Science, Naresuan University, Phitsanulok 65000, Thailand; sararatm@nu.ac.th (S.M.); sukunyaj@nu.ac.th (S.R.); 3 Department of Pharmaceutical Technology, Faculty of Pharmaceutical Sciences and Center of Excellence for Innovation in Chemistry, Naresuan University, Phitsanulok 65000, Thailand; jarupav@nu.ac.th

**Keywords:** N-vinylformamide, cationic hydrogels, poly(vinyl amine), potassium azeloyl diglycinate, pH-responsive release

## Abstract

This study explores the synthesis and modification of poly(N-vinylformamide-co-N-hydroxyethyl acrylamide) (poly(NVF-co-HEA)) hydrogels for cosmetic applications. Poly(NVF-co-HEA) hydrogels were produced followed by an acid hydrolysis reaction to produce poly(NVF-co-VAm-co-HEA) hydrogels, introducing poly(vinyl amine) (PVAm) into the structure. This modification considerably alters the hydrogels’ properties, yielding materials with over 96% water content, predominantly in the form of non-freezing or free water, which is beneficial in the uptake and release of hydrophilic species. The primary amine groups from inclusion of VAm also improved the mechanical properties, as evidenced by an 8-fold increase in Young’s modulus. The hydrogels also possessed pH-responsive behavior, which was particularly noticeable under acidic and basic conditions, where a large decrease in water content was observed (40% to 75% reduction). Characterizing the hydrogels’ release capabilities involved using organic dyes of different functional groups and sizes to examine the pH impact on release. The results indicated that hydrolyzed hydrogels interacted more effectively with charged species, highlighting their suitability for pH-responsive delivery. The release of cosmetic active ingredients was also demonstrated through the controlled release of Liquid Azelaic™, specifically potassium azeloyl diglycinate (PAD). Our findings reveal that the hydrolyzed hydrogels exhibit superior burst release, especially under alkaline conditions, suggesting their suitability for cosmetic applications where controlled, pH-responsive delivery of active ingredients is desired. Overall, this investigation highlights the potential of hydrolyzed poly(NVF-co-HEA) hydrogels in cosmetic applications. Their ability to combine high water content with mechanical integrity, along with their pH-responsive release ability, allows for use in cosmetic formulations.

## 1. Introduction

Hydrogels have become a compelling option within the field of advanced materials, offering versatility across biomedical, environmental, and industrial applications [1,2,3]. Hydrogel technology has found a remarkable niche within the cosmetic industry, particularly in the form of facial masks [4]. These masks leverage the distinctive properties of hydrogels to deliver a range of skincare benefits. However, their performance and consumer acceptance are hindered by a few challenges, notably the scarce variety of cationic materials used in their formulation. Predominantly, hydrogels are made with anionic or nonionic polymers, which may limit their interaction with the negatively charged components of the skin, potentially diminishing product efficacy. Cationic materials, often referred to as positively charged ions or molecules, are used in the cosmetics industry for various applications, offering numerous benefits. For example, they can enhance moisture retention and ensure active ingredients adhere better to the skin for improved absorption [5]. When considering cationic polymers, chitosan is frequently used. However, its use in cosmetics and various other fields is hindered by its water insolubility [6]. In contrast, synthetic cationic polymers have demonstrated significant potential in medical applications, such as drug delivery [7], gene therapy [8], and tissue engineering [9]. The primary challenge with natural polymers lies in their batch-to-batch variability. Thus, synthetic cationic polymers provide significant advantages, including customizable structures that improve biocompatibility, stability, and performance. Their development aims to broaden the range of options for formulators, meeting the increasing demand for more specialized and effective cosmetics [10].

Poly(vinylamine) (PVAm) is an attractive synthetic polycation due to its high nitrogen content, which gives it a high reactivity of primary amines. From this perspective, PVAm stands out from conventional polycations such as poly(ethylenimine) and poly(allylamine). Currently, the literature does not feature the widespread use of PVAm gels, with the majority of PVAm uses being in the paper industry [11,12,13]. This restricted utilization may stem from the challenge that poly(vinylamine) cannot be directly synthesized from the vinylamine monomer, owing to the monomer’s low stability and its inclination to tautomerize into acetaldehyde imine [14]. A more appealing strategy consists of the polymerization of N-vinylformamide (NVF), the structurally simplest vinyl amide monomer [15], followed by hydrolysis of the resulting poly(N-vinylformamide) (PNVF) in an aqueous acidic or basic medium [16,17]. Alkaline hydrolysis results in complete conversion from amide to amine and can even hydrolyze the amide groups involved in the cross-linkage. The acidic hydrolysis on the other hand does not reach completion probably due to electrostatic reasons and the self-cross-linked gel structure is retained post hydrolysis [18].

In our previous work, we investigated a novel hydrogel system of a crosslinked copolymer from the monomers N-vinylformamide (NVF) and N-hydroxyethyl acrylamide (HEA). HEA, with its hydrophilic hydroxyl and amide groups, forms a polymer (PHEA) that is emerging as a valuable biomaterial for long-lasting antifouling properties and durability in biomedical applications [19,20,21]. The hydrogels made from poly(N-vinylformamide-co-N-hydroxyethyl acrylamide) (poly(NVF-co-HEA)) exhibited a high water content, exceeding 80%, and a significant proportion of free water, over 70%. This composition enables them to absorb and release hydrophilic substances efficiently [22]. An advantage of these hydrogels containing PNVF is that a previously mentioned NVF can be hydrolyzed to PVAm. For instance, the research conducted by Ajiro et al. includes several studies involving hydrolyzed NVF [23,24,25,26], such as the creation of a novel gradient hydrogels in the form of a surface polyion complex (sPIC) gel [27].

In this study, we explored how the hydrolysis of NVF influences the characteristics of the poly(NVF-co-HEA) hydrogel. To conduct a comprehensive analysis, a control hydrogel composed of PNVF and HEA was synthesized, designated as the non-hydrolyzed (NH_Hydrogel). A hydrogel with the same composition was also prepared and subsequently subjected to acid hydrolysis, resulting in what is referred to as the hydrolyzed gel (H_Hydrogel). The two hydrogels’ properties were analyzed by measuring the equilibrium water content (EWC), water structuring, pH stability, and swelling/de-swelling cycling. Next, the controlled release performance of the two hydrogels was examined. This was performed using two approaches, first, by using three dyes, wherein the selected dye molecules possess different sizes, functional groups, and partial coefficients. This enables the behavior of the hydrogels at different pH values, with different charge dependent functional groups, to be studied. The second release approach evaluated the pH-dependent release of a cosmetic active ingredient from the hydrogels. Liquid Azelaic™, a formulation of Azelaic Acid in the form of Potassium azeloyl diglycinate (PAD), served as the model cosmetic ingredient. PAD is utilized for skin brightening, dark spot reduction, oil control on the skin, pore oil production regulation, and acne prevention. Its effectiveness is attributed to the competitive inhibition of tyrosinase [28,29,30].

## 2. Results and Discussion

### 2.1. Preparation of Poly(NVF-co-VAm-co-HEA) Hydrogel

**Figure 1:**
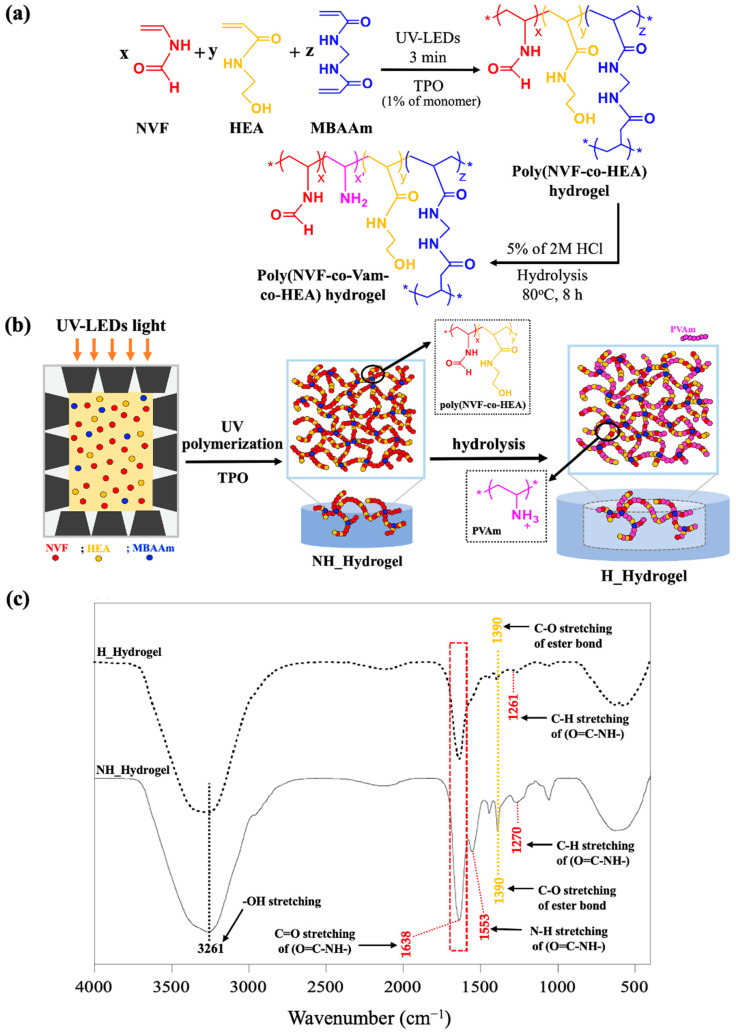
Preparation of hydrolyzed hydrogel (H_Hydrogel). (**a**) Schematic of preparation partway of poly(NVF-co-VAm-co-HEA) hydrogels in two steps: (1) preparation of poly(NVF-co-HEA) hydrogel and (2) hydrolysis to amino group. (**b**) Schematic illustration of preparation of H_Hydrogel. (**c**) FTIR Spectra of H_Hydrogel and NH_Hydrogel.

The hydrolyzed hydrogels (H_Hydrogel) were prepared in two steps as follows: (1) preparation of poly(NVF-co-HEA) hydrogels and (2) partial hydrolysis of the NVF pendent group within poly(NVF-co-HEA) hydrogels under acid conditions as shown in Figure 1a,b.

Following the polymerization of the hydrogels, the thickness of the dehydrated NH_Hydrogels was found to be approximately 0.1 cm. Initially, these dehydrated hydrogels appeared opaque, stiff, and brittle. However, after immersing them in DI water for 5 days—with daily water changes—they transformed into transparent, soft, and flexible gels. Subsequently, partial hydrolysis of the poly(NVF-co-HEA) hydrogels was carried out using a 5% solution of 2 M HCl, resulting in the formation of poly(NVF-co-VAm-co-HEA) hydrogels (H_Hydrogel). The hydrolysis process caused a visual difference with an increase in the dimensions of the hydrogels. To fully understand and describe this system, the properties of both the NH_Hydrogel and the H_Hydrogel were examined and compared. The chemical composition of the hydrogels was compared to demonstrate the success of the hydrolysis reaction. Figure 1c presents a comparison between NH_hydrogel and H_hydrogel samples. For NH_hydrogel, the characteristic peaks of C=O stretching, N–H stretching, and C–N stretching vibrations of the primary amide (O=CH–NH–) in the PNVF structure appeared at 1645 cm^−1^, 1530 cm^−1^, and 1280 cm^−1^, respectively [31]. Peaks at 1440 cm^−1^ and 1390 cm^−1^, associated with PHEA, were present in both samples. In the H_hydrogel sample, there is a noticeable reduction in the peaks associated with PNVF, particularly the carbonyl group (C=O) peak of the primary amide at 1645 cm^−1^.

### 2.2. Equilibrium Water Content (%EWC) and Water Structure

To help confirm the success of the hydrolysis reaction, NH_Hydrogel and H_Hydrogel were compared for any changes in their water profiles, with bulk water contents measured and reported as equilibrium water content (%EWC). Further analysis of each hydrogel’s water molecule behavior was conducted using differential scanning calorimetry (DSC), which helps differentiate between the two distinct states of water within hydrogels: freezing water (free water) and non-freezing water (bound water). The %EWC and quantities of freezing and non-freezing water in hydrophilic polymers depend on the density of hydrophilic groups present in the polymer. Consequently, these measures are valuable for distinguishing compositional differences among hydrogels.

**Figure 2:**
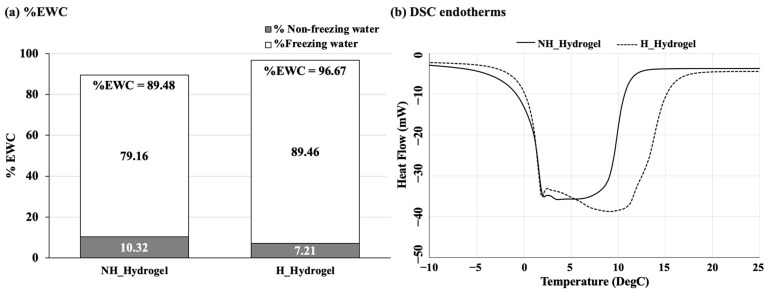
(**a**) %EWC and (**b**) DSC endotherms of NH_Hydrogel (poly(NVF-co-HEA) hydrogel) and H_Hydrogel (poly(NVF-co-VAm-co-HEA) hydrogel.

The water content (%EWC) of each sample was measured and is displayed in Figure 2a, while Figure 2b illustrates the endothermic peaks corresponding to the frozen water within the hydrogel. The area under these peaks represents the quantity of freezing water present. For the NH_Hydrogel, the %EWC is notably lower at 89.48%, with a small proportion of freezing water (10.32%), which results in a free-to-bound-water ratio of 8.7:1. For example, homopolymer hydrogels of HEA, carboxyethyl acrylate (CEA), and hydroxyethyl methacrylate (HEMA) have a free-to-bound-water ratio of 4.5:1, 1.3:1, and 1:8, respectively [22,32]. For the H_Hydrogel, the %EWC is higher at 96.67%, with an even smaller proportion of freezing water (7.21%) and a free-to-bound-water ratio of 13.4:1.

These results indicate that the inclusion of a hydrolysis reaction step modifies the water-binding characteristics of the hydrogel. This modification can be associated with the incorporation of primary amine groups from PVAm into the hydrogel. This incorporation suggests a movement towards more hydrophilic properties and interactions within the H_Hydrogel, enhancing its ability to retain and manage water in a state that does not freeze. The enhanced capacity for water retention, especially for water that does not freeze can be important in delivery applications. Moreover, an increase in the amount of free water in the hydrogels enhances their capacity to dissolve and absorb a larger quantity of hydrophilic substances.

### 2.3. Mechanical Properties

**Figure 3:**
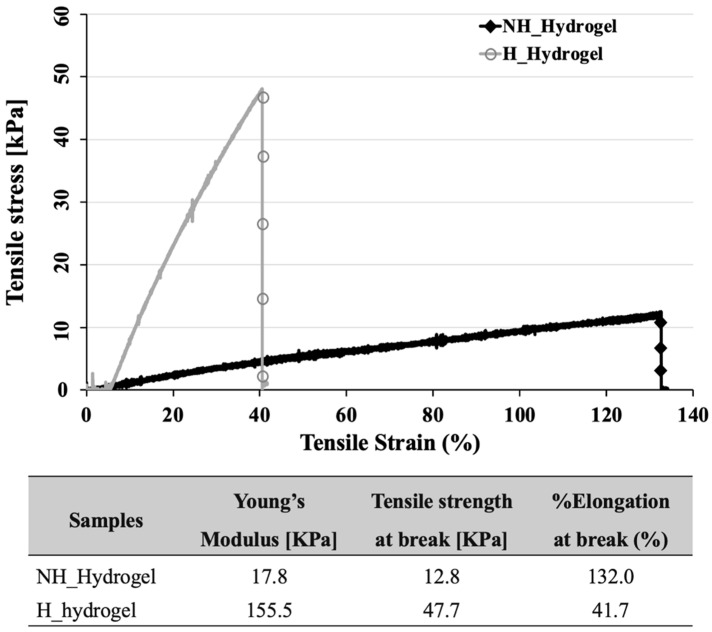
The stress–strain curves of pH of non-hydrolyzed hydrogels and hydrolyzed hydrogels.

To explore the differences between NH_Hydrogels and H_Hydrogels more thoroughly, we compared their mechanical properties. This involved examining their tensile properties, with the findings presented in Figure 3. Our analysis concentrated on key parameters: Young’s modulus (KPa), tensile strength at break (KPa), and %elongation at break (%E). These values collectively provide insights into the flexibility, strength, and stiffness of the hydrogel samples, offering a comprehensive view of their mechanical performance.

In comparing NH_Hydrogel with H_Hydrogel, considerable differences in their mechanical properties become apparent. H_Hydrogel exhibits a substantially higher Young’s modulus (155.5 KPa versus 17.8 KPa), greater tensile strength at break (47.7 KPa versus 12.8 KPa), but a lesser elongation at break (41.7% versus 132.0%). These distinct mechanical characteristics suggest that NH_Hydrogel is better suited for uses that demand elasticity and flexibility, while H_Hydrogel, due to its enhanced tensile strength and modulus, is preferable for applications needing a more rigid material capable of enduring greater stresses, albeit with reduced stretchability. The superior tensile strength and modulus observed in H_Hydrogel result directly from structural changes induced by the hydrolysis reaction. The increase in modulus is likely due to additional interactions within the hydrogel, now incorporating primary amine groups from PVAm. These interactions, including the hydrogen bonding with HEA and MBAAm, result in an approximately 8-fold (773%) increase in modulus, albeit with a 68% decrease in elongation. These interactions, like increased hydrogen bonding, are indicated by slight peak shifts in the FTIR spectra of H_hydrogel compared to NH_Hydrogel, as shown in Figure 1c. This observation aligns with studies in the paper industry, where PVAm has been utilized to improve mechanical properties, particularly in boosting wet adhesion between cellulose surfaces [33] and strengthening fragile paper [34].

### 2.4. Stability of Hydrogel

**Figure 4:**
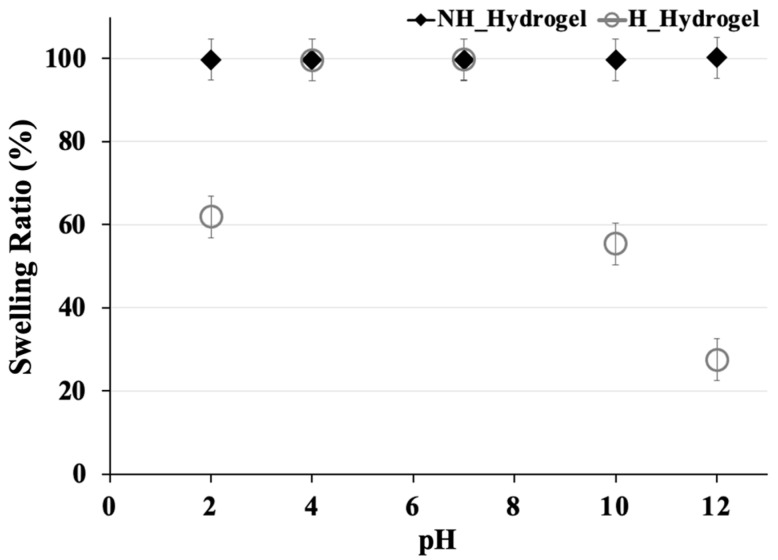
Stability comparisons of NH_Hydrogel and H_Hydrogel with the swelling behavior as a function of pH (error bars are ±SD, *n* = 3).

**Figure 5:**
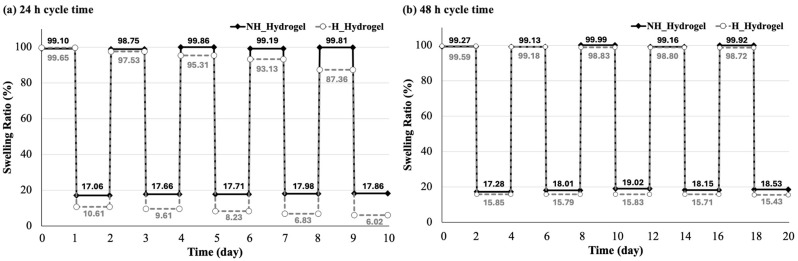
The swelling and de-swelling behavior of the hydrogels in water and ethanol, assessed through cycling tests that compare different durations: (**a**) a 24 h cycle time and (**b**) a 48 h cycle time.

Following the analysis of mechanical performance, our investigation examined the stability of NH_Hydrogels and H_Hydrogels under varying pH conditions (Figure 4), as well as their de-swelling behavior in ethanol and subsequent swelling in water through cycling tests (Figure 5). This aims to uncover how these hydrogels respond to environmental changes, offering insights into their stability and functional adaptability.

The results from Figure 4 clearly demonstrate that the NH_Hydrogel exhibits a stable swelling ratio throughout the tested pH range, achieving 100% of its EWC. This suggests that the network structure of the NH_Hydrogel remains consistent and largely unaffected by variations in environmental acidity or alkalinity. While after hydrolysis, the swelling behavior of this hydrogel varies more noticeably with pH, showing higher swelling ratios at specific pH values, the EWC of H_Hydrogel showed the maximum value at pH 7. At low pH (pH 2), electrostatic repulsive forces between like-charged regions may have reduce and collapse the polymer network. At high pH (pH 10 and 12), electrostatic interactions between oppositely charged regions may have occurred, along with the deprotonation of ammonia ions into amines in the polymer chain, causing the network to shrink. As a result, the water capacity decreased with further increases in pH (10, 12).

To understand the hydrogel swelling behavior and partitioning of solvents, the stability is quantified by the swelling ratio based on a reversible swelling and de-swelling process, which is expressed as a percentage by first looking at their repeated swelling and de-swelling cycles in two different solvents: water and ethanol. This was also conducted over two different time periods (24 h and 48 h). For the 24 h cycling (Figure 5a), the data indicate that NH_Hydrogel maintains a high swelling ratio in water over the five cycles, with percentages remaining around 99%. In contrast, the swelling ratio in ethanol is significantly lower, around 17–18%, but it remains relatively consistent across the cycles. This suggests that NH_Hydrogel is highly stable in water, maintaining its ability to swell to nearly its full capacity, while in ethanol, it swells much less but again with consistent performance over time. Similarly, the swelling ratios of H_Hydrogel (Figure 5a) also exhibits high stability in water, although there is a decrease in swelling ratio from 100% down to around 88%, while the swelling ratios in ethanol start at a lower value around 10% and decrease slightly over successive cycles, reaching around 4–5% by the fifth cycle, meaning that the gel does not have the ability to reach equilibrium in either water or ethanol. Therefore, the cycle time was increased to 48 h (Figure 5b) to give the H_Hydrogel more time to reach equilibrium. Within this time period, the H_Hydrogel maintains a high swelling ratio in water over the five cycles, with percentages remaining around 99%. The swelling ratio in ethanol also remains relatively consistent across the cycles; additionally, it reaches higher values than those in the 24 h cycle. Overall, both hydrogels represent the ability of hydrogels to re-equilibrate in water and ethanol, especially after 48 h. This information could be valuable for predicting the performance of these hydrogels in certain applications.

### 2.5. pH-Responsive Dye Release Behavior

**Figure 6:**
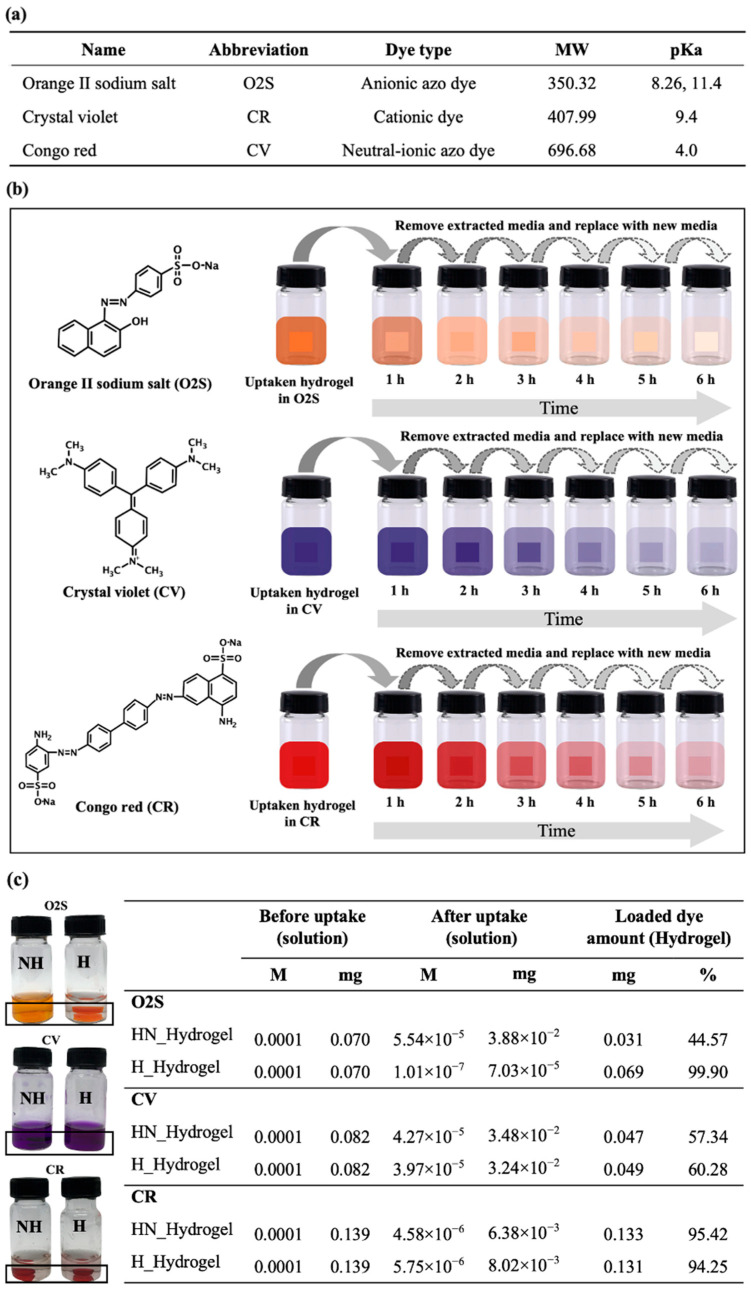
(**a**) Dye properties. (**b**) Dye structure (**left**) and schematic illustration of dye release methodology with orange II sodium salt (O2S), crystal violet, and Congo red from the hydrogel (**right**). (**c**) Visual comparison of non-hydrolyzed (NH) and hydrolyzed hydrogel (H) dye uptake capability with dye at pH 7 (**left**) and quantitative dye uptake amounts.

**Figure 7:**
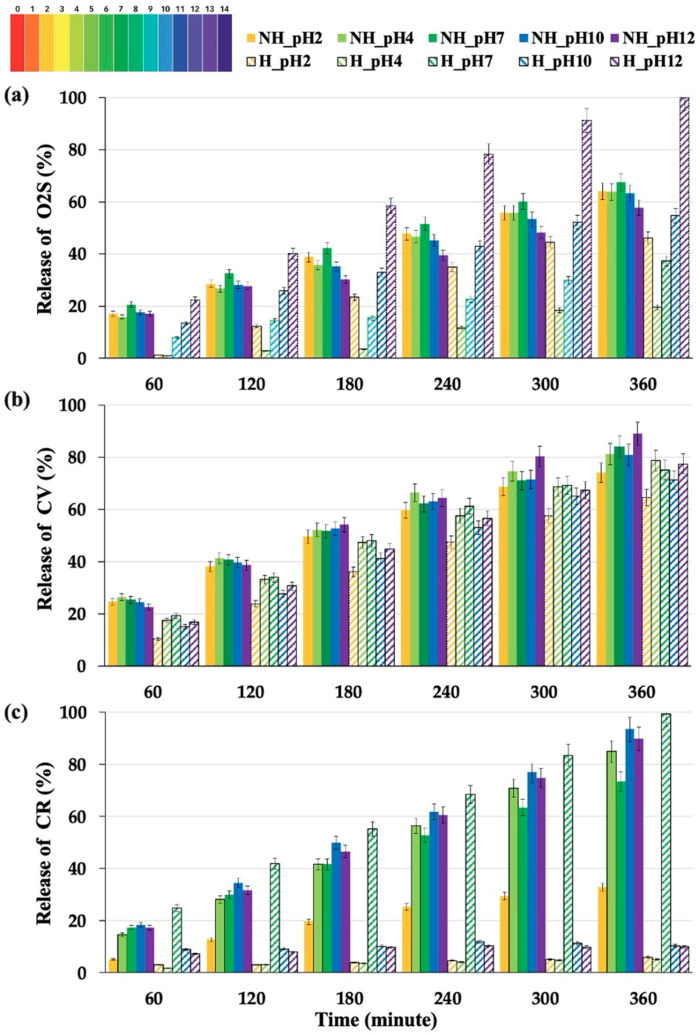
The release charts show the behavior of non-hydrolyzed and hydrolyzed hydrogels with (**a**) Orange II sodium salt (O2S), (**b**) crystal violet (CV), and (**c**) Congo red (CR) under various pH conditions: pH 2, 4, 7, 10, and 12 (error bars are ±SD, *n* = 3).

As seen with the stability in different pHs the two gels behavior vary differently with the poly(NVF-co-VAm-co-HEA) hydrogel having the potential for pH response as seen from the reduction in swelling ratio at pHs of 2, 10, and 12. In order to further investigate how this different behavior will influence their release characteristics, three dyes were used for uptake and release. These entailed the use dye solutions (orange II sodium salt (O2S), crystal violet (CV), and Congo red (CR)), that were loaded into the hydrogels at room temperature. The chosen dyes, with their variety of functional groups and molecular weights (as shown in Figure 6a,b), were selected to facilitate an understanding of their release behavior under different pH conditions. Moreover, the quantity of dye used was intentionally kept low to discern any minor differences in release patterns. The subsequent release studies, illustrated in Figure 7, were conducted in DI water across various pH levels.

To initially examine the dye uptake capabilities of the hydrogels, observations were made on the color changes in the hydrogels pre- and post-dye uptake. The pictures of the sample vials in Figure 6c compare the in vitro uptake of NH_Hydrogel (NH) and H_Hydrogel (H) at a neutral pH level with the three dyes. The assessment of dye uptake was further quantified by measuring the UV absorption of the dye solutions before and after the uptake process. According to the data in Figure 6c, both hydrogels demonstrated varying degrees of dye adsorption. Specifically, the NH_Hydrogel and H_Hydrogel exhibited loading percentages of 44.57% and 99.90% for the O2S dye, respectively. For CV dye, the absorption rates were 57.34% for NH_Hydrogel and 60.28% for H_Hydrogel. In the case of CR dye, the uptake was 95.42% for NH_Hydrogel and 94.25% for H_Hydrogel. These findings indicate a notable color change in each hydrogel, depending on the type of dye absorbed. H_Hydrogel showed a complete absorption of the anionic O2S dye, which can be attributed to the ionic interactions between the cationic functional groups in PVAm within the hydrogel and the anionic groups in O2S. Both NH_Hydrogel and H_Hydrogel demonstrated strong adsorption capabilities for CR dye. The pKa values of the dyes, as shown in Figure 6a, play a pivotal role in determining the pH levels at which the dyes are prone to protonation or deprotonation, influencing their charge and solubility. These values are essential for understanding the mechanisms behind the dyes’ retention or release by the hydrogels.

Figure 7 illustrates the cumulative percentage of dye release from NH_Hydrogel and H_Hydrogels over time, monitored across various pH conditions (pH 2, 4, 7, 10, and 12). The release charts show notable differences between NH_Hydrogel and H_Hydrogels, especially with O2S dye (Figure 7a). NH_Hydrogel exhibits a consistent release across most pH levels, with a slightly lower release at pH 12. Conversely, H_Hydrogel, which contains PVAm, demonstrates pH-responsive release capabilities, with lower release rates at acidic and neutral pH levels and significantly higher release at basic pH levels (pH 10 and 12), with pH 12 showing a release rate 60% higher than that of any other sample. This stems from the pKa values of primary amines typically range from about 9 to 11. This means that at a pH lower than the pKa, the amine group is mostly protonated (NH_3_^+^) carrying a positive charge and capable of electrostatic interactions with negatively charged groups. For CV dye release (Figure 7b), the release rates for NH_Hydrogel and H_Hydrogels are similar, with both hydrogels showing no significant change in release profile across different pH levels. CV contains tertiary (R_3_N) amines, which have similar or slightly lower pKa values than do primary amines but follow the same principle in that they are protonated and positively charged at pH values below their pKa. Thus, it is unlikely that there will be strong interactions with either hydrogel.

Regarding CR dye release (Figure 7c), a pronounced difference is observed in the release behavior between NH_Hydrogel and H_Hydrogels. NH_Hydrogels maintain a consistent release profile under varying pH conditions, with a minor decrease in CR release at pH 2. H_Hydrogels, however, display a predominately low CR release, with the highest release noted at pH 7. The release at pH 7 can be attributed to the uptake methodology, which is carried out at this pH, and therefore, this release is diffusion driven. The behavior at the other pHs can be attributed to the CR structure, which includes both sulfonic acid and amine groups, enabling interactions with all three polymers present in the hydrogels. Previously, we discussed the role of primary amine groups. It is also noteworthy that the sulfonic acid groups in CR are considered strong acids, characterized by pKa values typically below 1. Thus, CR and H_Hydrogels demonstrate intricate interactions across a broad spectrum of pH levels. Furthermore, at pH 12, H_Hydrogels undergo collapse, and given that CR has the highest molar mass among the dyes tested—71% greater than CV (407.99 for CV vs. 696.68 for CR)—this phenomenon could lead to the dye being entrapped within the contracted hydrogel structure.

### 2.6. Release of Cosmetic Active Ingredient (Potassium Azeloyl Diglycinate (PAD))

To study the release behavior of a cosmetic active ingredient from the poly(NVF-co-VAm-co-HEA) hydrogel in relation to pH changes, Liquid Azelaic™ was chosen as the model substance. This product, a derivative of azelaic acid known as potassium azeloyl diglycinate (PAD), served as the test cosmetic ingredient. A 1.5%wt solution of PAD was incorporated into the hydrogel following the same procedure used for the dyes. To analyze the uptake behavior of the hydrogels, Table 1 presents a comparison of the amounts of PAD absorbed by both NH_Hydrogel and H_Hydrogel at pH 7.

According to the data in Table 1, both hydrogels demonstrated varying degrees of PAD adsorption. Specifically, the NH_Hydrogel and H_Hydrogel exhibited loading percentages of 44.11% and 29.57% for PAD, respectively. It is important to highlight that the amount of PAD uptake significantly exceeds that of the dye uptake shown in Figure 6c. This is because the cosmetic active ingredient is utilized at concentrations typical of commercial applications.

**Figure 8:**
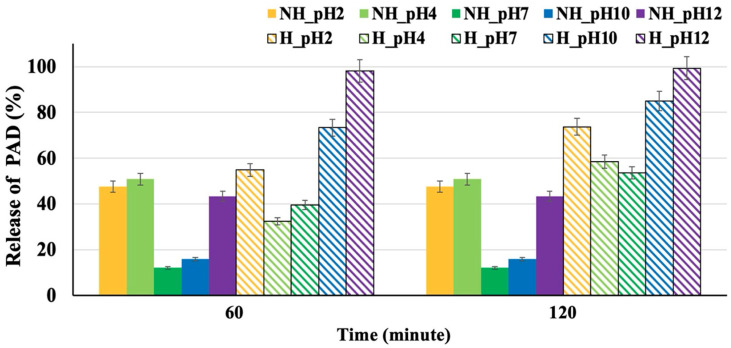
pH-responsive release charts for potassium azeloyl diglycinate (PAD) across various pH levels (error bars are ±SD, *n* = 3).

The cumulative percentage of PAD released from the hydrogels at different pH values over 2 h (120 min) is shown in Figure 8. The data reveal that the PAD release from NH_Hydrogel follows a burst release pattern, followed by very little subsequent release over the remaining 4 h (data presented in the Supplementary Materials; Figure S1). Under acidic conditions (pH 2 and 4), around 50% of the PAD contained in the NH_Hydrogel is released. However, at pH 7 and 10, the release rates drop to less than 20%, and at pH 12, the release is around 40%. For the H_Hydrogel, a burst release pattern is observed again, but the hydrolyzed gel can still release PAD from its structure in the second hour. Under acidic conditions (pH 2 and 4), the total release amounts to 70–75%. At a neutral pH of 7, the release is about 55%, and at higher pH levels (pH 10 and 12), the release rate increases to 90–100%. This behavior is attributed to the H_Hydrogels tendency to collapse at these pH values, with the greatest collapse occurring at pH 12, which in turn allows for the expulsion of more PAD from the hydrogel system. To better understand the release kinetics, three release kinetic models were used to assess cumulative release: zero-order (cumulative release vs. time), first-order (log cumulative release vs. time), and Higuchi’s (cumulative release vs. square root of time) [35,36]. The data suggest that the release from the hydrogel samples studied was best described by Higuchi’s model (data presented in the Supplementary Materials, Table S1).

## 3. Conclusions

In this study, we produced poly(NVF-co-HEA) hydrogels and subsequently modified them through acid hydrolysis to yield poly(NVF-co-VAm-co-HEA). The combination of NVF, HEA, and VAm produced hydrogels with several notable characteristics. The acid-hydrolyzed hydrogels exhibited exceptionally high water content, exceeding 96%, of which approximately 90% was either non-freezing or free water. The inclusion of VAm also considerably altered the mechanical performance of the hydrogels, with an 8-fold increase in Young’s modulus. Furthermore, we assessed the stability of the gels, with the hydrolyzed hydrogels providing pH-responsive behavior, with a reduction in water content under both acidic (pH 2) and basic (pH 10 and 12) conditions. The release patterns of three organic dyes, varying in functional groups and molecular sizes, were also examined across different pH levels to understand the hydrogels’ differential behavior. Notably, charged species exhibited distinct interactions with the hydrolyzed hydrogels. The study showed that release from the hydrogel matrices decreases over time, most fitting the Higuchi’s model. In summary, the introduction of the primary amine groups from VAm offers additional pH-responsive capabilities and mechanical strength over the non-hydrolyzed hydrogels. The hydrolyzed hydrogels were also able to release more cosmetic active ingredient than the non-hydrolyzed hydrogel, highlighting their potential application in cosmetic hydrogel formulations.

## 4. Materials and Method

### 4.1. Materials

N-vinylformamide (NVF, 98%), and N-hydroxyethyl acrylamide (HEA) (monomers), N,N′-methylenebisacrylamide (MBAAm) (crosslinker), diphenyl (2,4,6-trimethylbenzoyl) phosphine oxide (TPO) (photo-initiator), and phosphate-buffered saline (PBS) were all purchased from Sigma-Aldrich Co., Inc., Singapore, with all chemicals used as received. Hydrochloric acid (HCl) was purchased from by Ajax Finechem, Sydney, Australia. For the drug release procedure, the following organic dyes were used: Orange II sodium salt (O2S), Crystal violet (CV), and Congo red (CR), all purchased from Sigma-Aldrich Co., Inc., Singapore. Liquid Azelaic™ (Potassium azeloyl diglycinate), used as the cosmetic active ingredient, was purchased from MySkinRecipes, a brand of Chanjao Longevity Co., Ltd., Bangkok, Thailand.

### 4.2. Preparation of Hydrolyzed Hydrogels (H_Hydrogel) Based on Poly(NVF-co-HEA)

The poly(NVF-co-HEA) (75:25) hydrogels were first prepared by UV-LED photo-polymerization following our previously established methodology [22]. Briefly, the hydrogels for this study were prepared using free-radical polymerization using 1 wt% of photo-initiator (PI) and 0.5 wt% N,N-methylenebisacrylamide (MBAAm) as crosslinker. The dry dimensions of the hydrogel were controlled by injecting 1.5 mL of the hydrogel mixture between two glass plates lined with PET sheets to prevent adhesion after polymerization. The mold size had a width of 4 cm, a height of 4 cm, and a thickness of 0.1 cm. The mold was placed under UVLEDs for 2 min to cure the mixture into a crosslinked polymer network (UV-LED light source, emitting at a wavelength of 395 nm with an intensity of 80.0 mW cm^−2^). The polymer sheets were then removed from the mold and rinsed with deionized water (DI water) to remove any unreacted monomer and soaked in DI water for 5 days with the water being changed every day.

For the hydrolyzed hydrogels (H_Hydrogel), the poly(NVF-co-HEA) (75:25) hydrogel (NH_Hydrogel) was synthesized as above with the following additional hydrolysis reaction. The reaction used 5% of 2 M hydrochloric acid added to 250 mL of DI water in a three-neck round-bottom flask. Then, the NH_Hydrogel was placed into the round-bottom flask and refluxed at a temperature of 80 °C for 8 h to produce the H_Hydrogel. After hydrolysis, the H_Hydrogels were rinsed with phosphate-buffered saline solution (pH 7.4) and then immersed in DI water for 5 days, with daily water changes, to remove any residual unreacted material.

### 4.3. Characterization of Hydrogels

In addition, the fundamental hydrogel properties, or “base gel” properties, including chemical composition, equilibrium water content (%EWC), state of water, mechanical properties, and the stability of hydrogels, were first assessed to characterize the hydrogels. Regarding pH-responsive release, this was investigated to understand the ability of NH_Hydrogel and H_Hydrogel for active delivery.

#### 4.3.1. Chemical Composition (FTIR)

Fourier transform infrared (FT-IR) spectra were recorded on hydrogel samples before and after modification at room temperature (16 scans accumulated per spectrum) to characterize the functional groups by using a PerkinElmer Spectrum Two spectrometer (Waltham, MA, USA) with UATR at 4000–400 cm^−1^. All samples were kept at room temperature before testing.

#### 4.3.2. Equilibrium Water Content (%EWC)

The hydrogels were dehydrated by placing each hydrogel sample in a microwave for 2 min. Subsequently, the dehydrated gels were weighed and then returned to the microwave until their weight reached a constant value. Each hydrogel was measured three times under the same conditions, and the average values were reported (*n* = 3). The equilibrium water content was calculated according to the formula in Equation (1):

%EWC = ((W_w_ − W_d_)/W_w_) × 100% (1)

where W_w_ and W_d_ are the hydrated and dehydrated weights of the hydrogel, respectively.

#### 4.3.3. Differential Scanning Calorimetry (DSC)

The differentiation between freezing (free) and non-freezing (bound) water within the hydrogels was determined using differential scanning calorimetry (DSC) (Mettler Model DSC1, Greifensee, Switzerland). For this study, the hydrated hydrogels were sectioned, and each piece was weighed to around 10 mg. Excess surface water was gently removed with moist filter paper to avoid water uptake from the sample. This procedure was performed in triplicate for each sample (*n* = 3). The samples were then weighed again and enclosed in an aluminum pan to inhibit water loss through evaporation. These pans were placed in the thermal analyzer’s sample holder. The scanning was conducted under specific parameters:

- Cool from 25 °C to −70 °C at 20 °C/min
- Hold for 5 min at −70 °C
- Heat from −70 °C to −25 °C at 20 °C/min
- Heat from −25 °C to 25 °C at 10 °C/min

The energy needed to melt the freezing water within the sample is represented by the area under the endothermic peaks. Given the known weight of each sample and the energy required to melt 1 g of pure water (333.5 J·g^−1^) [37], the percentage of freezable (free) water in the sample can be calculated using Equation (2).

Freezing water (%) = [ΔH_Tr_/(m × ΔH_f_)] × 100 (2)

where ΔH_f_ = 333.5 J/g, ΔH_Tr_ = heat of transition, and m = sample weight (mg). The quantity of non-freezing water was determined by deducting the amount of freezing water from the total water content percentage, whereby:

Non-freezing water content (%) = Total water content (%) − Freezing water content (%)(3)

#### 4.3.4. Tensile Properties

Mechanical properties were determined by testing the hydrated hydrogels using a Universal Testing Machine (INSTRON^®^ CALIBRATION LAB model 5965 Norwood, MA, USA) to measure the tensile properties of the hydrogel samples. The tested hydrogel was cut into a rectangular shape measuring 20 mm × 60 mm, and each sample was repeated in triplicate (*n* = 3). The thickness of each patch was determined by averaging three separate measurements taken along the middle 20 mm section of the hydrogel using a micrometer. The hydrogel was clamped between two grips equipped with a 100 N load cell and then pulled at a rate of 20 mm/min until the samples ruptured. In this experiment, three specimens were tested for each hydrogel sample formulation. The Young’s modulus, tensile strength at break, and percentage of elongation at break of the hydrogels were reported.

#### 4.3.5. Stability of Hydrogel

##### Swelling Studies in Water–Ethanol Cycles

Swelling behavior in water–ethanol cycles was assessed using a standard gravimetric technique. The process began with weighing the fully hydrated hydrogels at their equilibrium water content (W_0_) after being left for specific durations, either 24 or 48 h. A 70% ethanol solution was then prepared, maintaining a consistent volume concentration of ethanol. The hydrogels were submerged in this ethanol solution for the same duration (24 or 48 h). After each immersion period, the weight (W_t_) of the hydrogels was recorded. Utilizing Equation (4), the hydrogels were alternately exposed to water and ethanol solutions across five cycles to assess the swelling behavior over these intervals. All tests were conducted at ambient room temperature.

Swelling ratio (%) = [(W_0_ − W_t_)/W_0_] × 100 (4)

where W_0_ is the weight of the completely hydration hydrogel, and W_t_ is the weight of hydrogel after the set time period (24 h or 48 h).

##### Swelling Studies as a Function of pH

For determining the swelling behavior as a function of pH of the hydrogel, a fixed amount of the hydrogel was observed at pH 2, 4, 7, 10, and 12 at room temperature (25 °C). First, three fully hydrated hydrogels at their full equilibrium water content from each composition were weighed (W_0_). Then, the hydrated hydrogel samples were immersed in desired pH solution and given enough time to reach equilibrium (48 h), and then weight (W_t_) was recorded, following Equation (4).

#### 4.3.6. Controlled Release System Experiment

To study the pH-responsive dye release behavior of hydrogels, three organic dyes and one cosmetic active ingredient were assessed (O2S (λ = 480 nm) [38], CV (λ = 590 nm) [39], and CR (λ = 495 nm) [40] were used as drug models), while potassium azeloyl diglycinate (PAD) was used as the cosmetic active ingredient. Dye uptake was carried out by immersing the NH_Hydrogel and H_Hydrogel in 2.0 mL of 0.0001 M solution of the three organic dyes and 1.5% wt of PAD for 96 h to allow the hydrogels to reach equilibrium. The loaded hydrogels were then placed into clean vials, each containing 1 mL of aqueous solution at various pH levels (pH 2, 4, 7, 10, and 12), and vortexed using a Vortex Mixer GENIE 2 (Model G560E, Bohemia, NY, USA) at 5000 rpm for 15 s. This process was repeated for six 60 min periods, resulting in a total release time of 6 h. After each time period, the solution was removed, and fresh pH aqueous solution was added to the vial for the next time period. The release percentages were determined and compared to the amount of dye loaded in the hydrogels, with each release performed in triplicate. UV absorbance values were measured using a multiplate reader (Biotek, Model Synergy H1 Hybrid Reader Santa Clara, CA, USA) and calculated to dye amounts using standard calibration curves for each dye solution.

For the determination of the PAD release, which is a derivative of azelaic acid (AA), we used the UV spectrophotometric method adopted for the determination of AA in pharmaceutical formulations as referenced in the work of Kishore et al. [41]. In summary, the method involves production of a stock solution containing, 1.0 mL PAD solution (1.5% wt) and 1.0 mL of pH 9.8 buffer and 0.5 mL of crystal violet. The total volume of the aqueous phase was then adjusted to 10.0 mL with distilled water. Finally, 10 mL of chloroform was added, and the contents were shaken for 2 min and allowed to separate. The organic layer was collected through a cotton plug, and the UV absorbance was immediately measured using a multiplate reader (Biotek, Model Synergy H1 Hybrid Reader Santa Clara, CA, USA) at 655 nm against a blank reagent.

## Figures and Tables

**Table 1:** Amount of PAD loaded into NH_Hydrogel and H_Hydrogel before and after the uptake process.

	Before Uptake (Solution)		After Uptake (Solution)		Loaded PAD Amount (Hydrogel)	
	%wt	mg	%wt	mg	mg	%
**PAD uptake**						
NH_Hydrogel	1.50	30.00	0.84	16.77	13.23	44.11
H_Hydrogel	1.50	30.00	1.06	21.13	8.87	29.57

## Data Availability

The raw/processed data required to reproduce these findings cannot be shared at this time as the data also forms part of an ongoing study.

## Supplementary Materials

The following supporting information can be downloaded at: https://www.mdpi.com/article/10.3390/gels10050311/s1, Figure S1: pH-responsive release charts for potassium azeloyl diglycinate (PAD) across various pH levels for 6 h (Error bars are ±SD, *n* = 3); Table S1: Linear correlation obtained from different kinetic models.
