# Supplementary material for: Characterization and Performance Analysis of Hydrolyzed versus Non-Hydrolyzed Poly(NVF-co-HEA) Hydrogels for Cosmetic Applications
Source: Gels. 2024 May 2;10(5):311. doi: 10.3390/gels10050311 (PMC11120761; doi:10.3390/gels10050311)
Supplement: Supplementary file 1 [file gels-10-00311-s001.zip › gels-2985365-supplementary.pdf]

Characterization and Performance Analysis of Hydrolyzed versus Non-Hydrolyzed Poly(NVF-co-HEA) Hydrogels for Cosmetic Applications

Maytinee Yooyod [1], Thanyaporn Pinthong [1], Sararat Mahasaranon [2], Jarupa Viyoch [3], Sukunya Ross [2] and Gareth M. Ross [2,*]

Electronic Supporting Information (ESI)

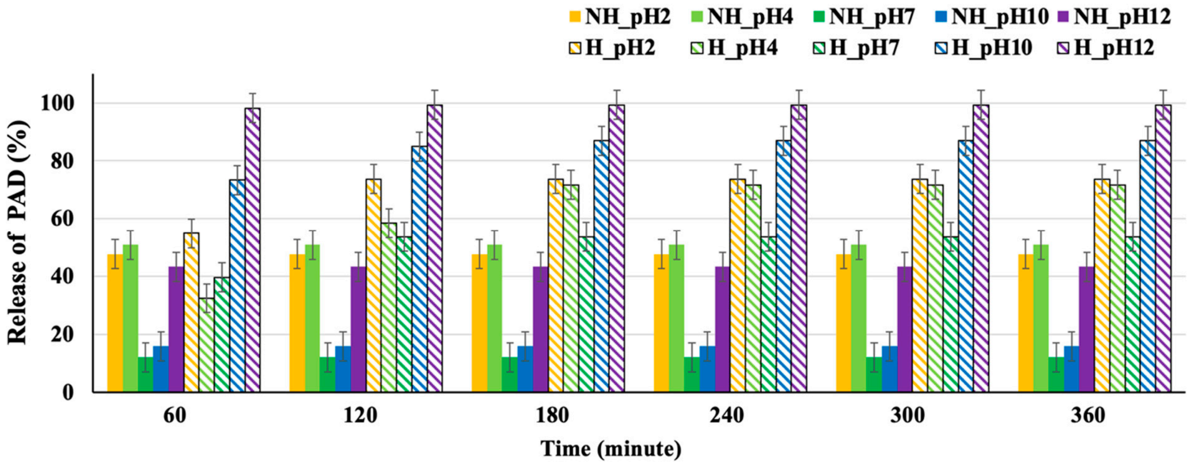

Figure S1. pH-responsive release charts for potassium azeloyl diglycinate (PAD) across various pH levels for 6 hours (Error bars are ±SD, n = 3).

Figure S1, shows the cumulative percentage of PAD released from the hydrogels at different pH values over 6 h (360 mins). The data reveals that the PAD release from NH_Hydrogel is burst release, followed by very little subsequent release over the remaining 5 hours. The correlation coefficients obtained from the various kinetic models are presented in Table S1. The data suggest that the release from the hydrogel samples studied was best described by Higuchi's model.

Table S1. Linear correlation obtained from different kinetic models

	Linear correlation (R^2)		
	**Zero-order**	**First-order**	**Higuchi**
**NH_Hydrogel**			
Average	0.751	0.750	0.919
**H_Hydrogel**			
Average	0.892	0.798	0.959
